# The Impact of Oral Whey Protein and Yeast Protein Supplementation for 6 Months on Skeletal Muscle Mass, Strength, and Function in the Elderly: A Randomized, Controlled, Parallel Study

**DOI:** 10.1002/fsn3.71552

**Published:** 2026-03-13

**Authors:** Bai Huijing, Shen Lei, Zhao Fang, Li Si, Tan Li, Gong Qinqing, Gu Dongmei, Sun Jianqin, Ma JiangChen

**Affiliations:** ^1^ Department of Nutrition Huadong Hospital, Fudan University Shanghai China; ^2^ Caohejing Community Health Center, Xuhui District Shanghai China; ^3^ Shanghai Elderly Nutrition and Health Quality Control Center Shanghai China; ^4^ Department of Geriatrics Affiliated Hangzhou First People's Hospital Chengbei Campus, School of Medicine, Westlake University (Hangzhou Geriatric Hospital) Hangzhou China

**Keywords:** muscle strength, physical function, skeletal muscle mass, whey protein, yeast protein

## Abstract

This study aimed to evaluate the effects of 6‐month daily supplementation with whey protein (WP) or yeast protein (YP) on anthropometric measurements, laboratory parameters, skeletal muscle mass, muscle strength, and physical function in Chinese older adults. In this randomized controlled trial, 92 participants were assigned to WP (*n* = 31), YP (*n* = 30), or control (*n* = 31) groups. At baseline and 24 weeks, we assessed: anthropometrics (weight, BMI, and waist/calf circumference); laboratory parameters (ALT, TG, TC, creatinine, urea, hemoglobin, and FBG); appendicular skeletal muscle mass (index); handgrip strength; and physical function (6‐m walk speed, chair stand test, SPPB). Habitual diet and activity were maintained. Anthropometrics: Compared to controls, both protein groups significantly reduced body weight (WP: −5.4 kg; YP: −0.9 kg; both *p* < 0.05). Laboratory Parameters: TC decreased significantly from baseline in both WP (−0.15 mmol/L) and YP (−0.18 mmol/L) groups (*p* < 0.05). Muscle mass and strength: No intergroup differences were found in changes of ASM or handgrip strength (*p* > 0.05). Physical function: Both intervention groups showed greater improvement in 6‐m walk speed and SPPB scores versus control (*p* < 0.05). The mean increase in SPPB score in the YP group (0.9 points) reached the threshold for a minimal clinically important difference. Six‐month supplementation with either WP or YP improved body weight, cholesterol, and physical function in Chinese older adults, with comparable effects between the two protein sources. YP may serve as a viable alternative to WP for enhancing functional status, a finding with potential clinical relevance.

## Graphical abstract

Daily 20 g whey or yeast protein for 6 months similarly improved physical function (SPPB +0.9 points; gait speed +0.1 to 0.2 m/s) and reduced body weight versus control in 92 community‐dwelling seniors (73 year). Muscle mass and grip strength remained unchanged. Yeast protein offers a cost‐effective, sustainable alternative to whey for maintaining mobility and lowering cardiometabolic risk in the elderly.

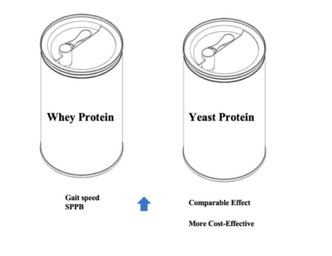

## 1 Introduction

Accelerated global population aging has made the progressive decline in skeletal muscle mass and associated physical functional deterioration among older adults a major public health concern. Sarcopenia, as a classic geriatric syndrome, not only significantly increases fall risk, limits mobility, and leads to loss of independence, but also exhibits a strong positive correlation with the occurrence rate of chronic disorders such as cardiometabolic diseases and osteoporosis, as well as all‐cause mortality (Chen et al. 2016, 2020; Cruz‐Jentoft et al. 2019; Habboub et al. 2025; Zanker et al. 2023). Of particular concern is that sarcopenia substantially elevates healthcare expenditures and overall medical burden (Darvishi et al. 2024). For instance, a UK study found that dynapenia (a key component of sarcopenia) was associated with markedly increased healthcare and social care costs, with annual additional medical expenses attributable to muscle weakness reaching £2.5 billion (Bruyère et al. 2019). In the United States, sarcopenia‐related hospitalization costs were estimated at $40.4 billion, with $19.12 billion incurred by adults aged ≥ 65 years (Goates et al. 2019). These data underscore the substantial economic burden of sarcopenia on healthcare systems, which is likely to worsen with population aging (Bruyère et al. 2019; Darvishi et al. 2024; Goates et al. 2019). Therefore, exploring effective and cost‐efficient interventions to delay or reverse this age‐related muscle decline is urgently needed.

Current management strategies for sarcopenia primarily include nutritional interventions, exercise training, pharmacological treatments, and lifestyle modifications. Of these, nutritional interventions—particularly protein supplementation—are critical for promoting muscle protein synthesis, maintaining muscle mass, and improving physical function. Whey protein, renowned for its high bioavailability and rich content of branched‐chain amino acids (BCAAs), has garnered widespread attention for enhancing muscle health (Kamińska et al. 2023; Liao et al. 2024; Nasimi et al. 2023).

Yeast protein (YP) (Baier et al. 2022; Timira et al. 2024), a microbial‐derived protein source, was approved as a novel food ingredient by China's National Health Commission on December 1, 2023 (Announcement No. 10, 2023). It is produced from *Saccharomyces cerevisiae* through cultivation, fermentation, centrifugation, and subsequent processing steps, including nucleic acid removal, enzymatic hydrolysis, extraction, purification, sterilization, and drying. Its primary nutritional components include protein (≥ 70.0 g/100 g), fat, dietary fiber, and moisture. Although international studies exist (Briskey et al. 2024), domestic research on its effects on skeletal muscle health in older adults remains limited. Currently, systematic comparisons between these two protein supplements regarding their impacts on body composition, metabolic parameters, muscle strength, and physical function in Chinese elderly populations are lacking.

This randomized controlled trial therefore aims to evaluate the effects of 6‐month supplementation with either whey protein (WP) or yeast protein (YP) among Chinese older adults. The primary outcomes will include anthropometric measurements (body weight, BMI, waist and calf circumference) and laboratory parameters (blood lipids, glucose, hepatic and renal function). It will also assess the effects on skeletal muscle mass, strength (handgrip), and physical function (6‐m gait speed, chair stand test, and Short Physical Performance Battery (SPPB) score). This study is expected to provide scientific evidence for optimizing protein supplementation strategies in this population.

## 2 Methods

### 2.1 Study Design and Grouping

**FIGURE 1:**
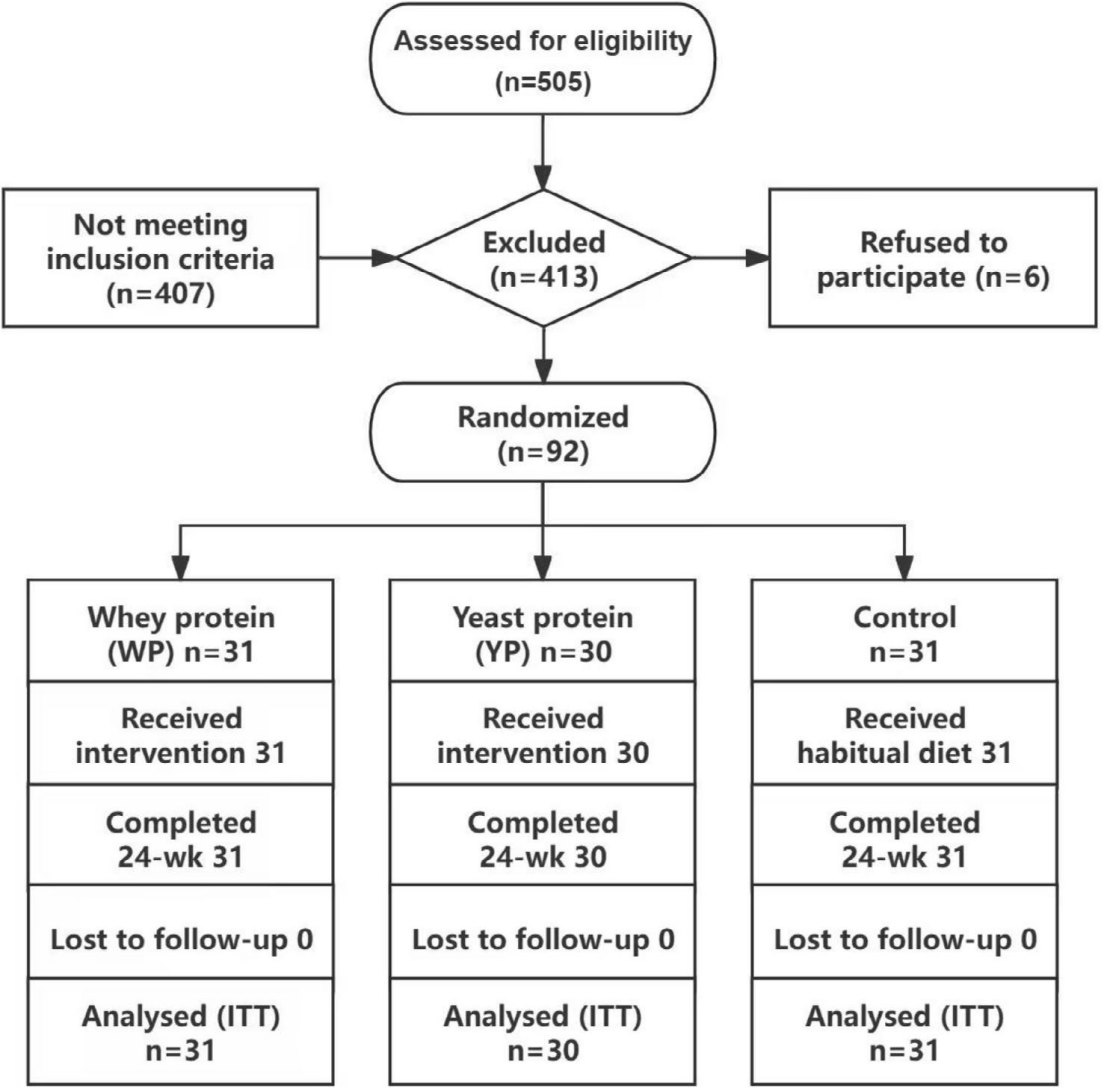
CONSORT 2020 flow diagram of study participants.

This study employed a prospective, randomized, controlled, parallel‐group design. After completing baseline assessments and providing written informed consent, participants were randomly assigned in a 1:1:1 ratio to the Whey Protein group (WP), Yeast Protein group (YP), or Control group by an independent statistician using a computer‐generated random number sequence (SAS 9.4 software, PROC PLAN procedure). To ensure allocation concealment, the randomization sequence and group assignments were stored in a centralized randomization system, accessible to the research team only after the aforementioned procedures were completed. Because of the physical nature of the interventions (daily consumption of protein powder beverages for the WP and YP groups versus no supplement for the Control group), blinding of participants was not feasible. However, to minimize assessment bias, all personnel responsible for measuring outcome indicators (including body composition, handgrip strength, gait speed, and SPPB assessments) were blinded to group assignments. Furthermore, data entry and statistical analysis personnel remained blinded until all analyses were completed. The two protein supplements (whey protein and yeast protein) were optimized in pre‐tests to be as similar as possible in packaging, color, odor, and taste, and were uniformly labeled and coded (A, B) by a third‐party supplier to reduce the likelihood of identification between the WP and YP groups. The WP group (*n* = 31) received a daily supplement of 20 g of whey protein (brand: Glanbia; protein content: 77.9%). The YP group (*n* = 30) received a daily supplement of 20 g of yeast protein (brand: Anpro; protein content: 80.0%). The Control group (*n* = 31) received no protein supplementation and maintained their usual diet. Detailed nutritional compositions of the yeast and whey proteins are provided in Table S1. Nutritional components of the protein powders were analyzed by the Hubei Provincial Key Laboratory of Yeast Function. The amino acid profiles of both protein powders were determined using an amino acid analyzer. Protein content was determined using the Kjeldahl method (GB 5009.5–2016), fat content using the acid hydrolysis method (GB 5009.6–2016), and amino acids using HPLC (GB 5009.124–2016). Three batches of the intervention products were analyzed, with three sachets randomly selected from each batch, and each sachet analyzed independently twice, covering all intervention product batches (coefficient of variation, CV < 5%). All participants were instructed to maintain their habitual dietary and physical activity patterns and to refrain from using additional protein powders, other nutritional supplements, or engaging in exercise specifically targeting skeletal muscle during the study period. To ensure protocol adherence, multiple compliance monitoring methods were employed: empty sachets were collected and counted against the number distributed at each 4‐week follow‐up; participants also completed daily intake logs (recording time and dose), which were verified on‐site by researchers; discrepancies between sachet counts and logs were immediately clarified and corrected with the participant. On the basis of this, the average daily number of protein sachets consumed was 1.90 and 1.99 for the WP and YP groups, respectively, with calculated compliance rates of 98.2% and 98.4%. The actual daily protein intake from the supplements was 15.23 ± 0.32 and 15.75 ± 0.43 g for the WP and YP groups, respectively. Additionally, tolerance to the supplement, dietary intake, and physical activity were recorded every 4 weeks. Participants were required to report any adverse events or physical discomfort to the researchers promptly for appropriate management and potential adjustment of the intervention. This study was registered on the Chinese Clinical Trial Registry (ChiCTR2200057116). The study protocol and informed consent form were approved by the Ethics Committee of Huadong Hospital Affiliated to Fudan University (2022 k010) prior to initiation. Inclusion criteria were: appendicular skeletal muscle mass (ASMI) measured by DXA < 7.0 kg/m^2^ for men and < 5.4 kg/m^2^ for women; age ≥ 60 years; ability to walk independently; and voluntary participation. Exclusion criteria included: severe liver or kidney disease, severe diabetes, severe hypertension, severe cardiovascular disease, cancer, infectious diseases, hyperuricemia, and gout; use of protein supplements within the past month; participation in high‐intensity resistance exercise within the past month; and hormone therapy within the past year. The CONSORT 2020 participant flow diagram is shown in Figure 1.

### 2.2 Intervention

The protein supplements were provided as powder in 10 g aluminum foil sachets. Participants were instructed to consume one sachet dissolved in 100 mL of warm water after breakfast and dinner each day. The intervention lasted for 24 weeks.

### 2.3 Measurements

All measurements were collected at baseline (0 months) and post‐intervention (24 weeks). For anthropometric measurements, body weight (kg) was measured using a calibrated electronic scale (model MSG003, Meilen, China), and height (m) was measured using a standard stadiometer (model MSG003, Meilen, China). Body mass index (BMI, kg/m^2^) was calculated as weight/height^2^. Waist circumference (cm) was measured at the umbilical level using a soft tape. Calf circumference (cm) was measured twice at the maximum circumference of the dominant leg, with the larger value used for analysis. For laboratory indicators, fasting blood samples were collected. Liver function—alanine aminotransferase (ALT, U/L); lipid metabolism—triglycerides (TG, mmol/L) and total cholesterol (TC, mmol/L); renal function—creatinine (Cr, μmol/L) and urea (Urea, mmol/L); and glucose metabolism—fasting blood glucose (FBG, mmol/L) were analyzed using a Hitachi 3500 automatic biochemical analyzer (Hitachi Instruments (Suzhou) Co. Ltd.). Hemoglobin (Hb, g/L) was determined using a Sysmex XM‐550 automated hematology analyzer (Sysmex Corporation). For nutritional and physical activity levels, dietary intake was assessed using a 24‐h dietary recall method, and nutrient supply was calculated using Shanghai SY Nutrition Analysis Software. Physical activity was assessed using the International Physical Activity Questionnaire (IPAQ), with results expressed as MET‐min/week. The validity of this questionnaire has been confirmed in Chinese populations and specific patient groups (Craig et al. 2003; Huang 2018; Li 2023). For skeletal muscle mass, ASM was measured using dual‐energy X‐ray absorptiometry (DXA, model Discovery Wi, Hologic Inc., USA). The appendicular skeletal muscle mass index (ASMI, kg/m^2^) was calculated as (ASM of left upper limb + ASM of right upper limb + ASM of left lower limb + ASM of right lower limb)/height^2^. The DXA scanner was initialized and calibrated by a professional technician prior to each test. The coefficient of variation for repeated measurements was 1.0%. In vivo precision errors (CV%) for the femoral neck, fat, muscle, hip, and lumbar spine were 1.86%, 1.5%, 0.74%, 0.95%, and 0.86%, respectively. For skeletal muscle strength, handgrip strength (kg) was measured using a handheld dynamometer (CAMRY EH101; GD, China) on the dominant hand. Three measurements were taken, and the maximum value was recorded. For skeletal muscle function, 6‐m gait speed (m/s) was calculated from the time required to walk 6 m at a usual pace. The chair stand test recorded the time required to complete five sit‐to‐stand cycles. The Short Physical Performance Battery (SPPB) (Eusepi et al. 2025) includes balance (0–4 points), gait speed (0–4 points), and chair stand test (0–4 points) components. The sum of these three scores yields the total SPPB score (0–12 points), with higher scores indicating better function: 0–6 points suggests poor muscle function, high fall risk, and potentially severe limitations in independent activity; 7–9 points indicates moderate muscle and physical function, representing a critical period for intervention; 10–12 points indicates good muscle function and physical capacity.

### 2.4 Quality Control

All surveys and measurements were conducted by uniformly trained and certified professional technicians.

### 2.5 Statistical Analysis

After verification for accuracy and completeness, data were entered into a database using Epidata 3.1. Statistical analyses were performed using IBM SPSS 26.0 software. All continuous variables are presented as mean ± standard deviation. The difference was defined as the post‐intervention value minus the baseline value. Between‐group comparisons for continuous variables were performed using analysis of variance (ANOVA) or the Kruskal–Wallis test, as appropriate. Categorical data were compared using the chi‐square test or Fisher's exact test. A *p*‐value < 0.05 was considered statistically significant. The sample size was estimated on the basis of the primary outcome, change in 6‐m gait speed. Referring to Kang et al. (Kang et al. 2019) and our pilot study (*n* = 20), the standard deviation (*σ*) for gait speed change was set at 1.29 m/min. Considering the study population consisted of older adults with pre‐sarcopenia and expecting a modest intervention effect, a conservative minimum detectable between‐group difference (*δ*) of 1.2 m/min (0.02 m/s) was set. Although this is lower than the minimal clinically important difference (MCID ≥ 0.05 m/s) proposed by Perera et al. (Perera et al. 2006), as an exploratory trial, priority was given to detecting smaller effects that might realistically occur. With a two‐sided *α* = 0.05 and 90% power (*β* = 0.10), approximately 24 participants per group were required. Accounting for a 20% dropout rate, 30 participants per group (total 90) were determined. This protocol was filed (ChiCTR2200057116). Although the mean International Physical Activity Questionnaire (IPAQ) scores showed minor fluctuations across the three groups during the intervention, the between‐group differences in these changes were not statistically significant (*p* = 0.478, Table 3). Furthermore, the magnitude of these fluctuations was less than 130 MET‐min/week, which is below the minimal detectable change (≈150 MET‐min/week) reported for the reliability of the Chinese version of the IPAQ (Huang 2018; Li 2023). Therefore, these minor differences are more likely attributable to measurement error rather than genuine changes in physical activity behavior. Additionally, the study protocol explicitly required participants to maintain their habitual physical activity levels. Compliance was monitored throughout the follow‐up period via empty package returns and log verification to confirm the absence of new structured exercise routines. For these reasons, the IPAQ score was not included in the primary analysis model to avoid over‐adjustment, which could bias the estimation of the protein intervention effect (Schisterman et al. 2009).

## 3 Results

A total of 92 older adults (mean age 73.2 ± 5.4 years; 35% male) were randomly assigned to the whey protein (WP, *n* = 31), yeast protein (YP, *n* = 30), or control (*n* = 31) group and completed the 6‐month follow‐up. At baseline, all measured characteristics—including demographic data, dietary intake, physical activity, body composition, muscle strength and function, anthropometrics, and blood biochemical parameters—were comparable across the three groups (all *p* > 0.05; see Table 1). No serious adverse events were reported. Adherence to the intervention was high, with rates of 98.2% in the WP group and 98.4% in the YP group.

**TABLE 1:** Basic characteristics analysis of all Chinese older adults in the study (*n* = 92).

	WP group (*n* = 31)	YP group (*n* = 30)	Control group (*n* = 31)	*p*
Age, years	73.4 ± 5.5	73.1 ± 6.0	73.0 ± 4.8	0.964
Sex, male/female	15/16	8/22	9/22	0.146
Dietary energy, kcal/d	1416.6 ± 490.1	1546.3 ± 618.1	1556.5 ± 249.3	0.698
Dietary carbohydrates, g/d	146.3 ± 48.7	171.6 ± 94.5	194.7 ± 42.2	0.202
Dietary fat, g/d	68.2 ± 37.4	69.5 ± 23.3	59.4 ± 11.0	0.656
Dietary protein, g/d	54.4 ± 2.7	58.7 ± 2.8	60.7 ± 1.2	0.707
Dietary protein, g/kg/d	0.953 ± 0.4	0.925 ± 0.3	0.933 ± 0.3	0.963
Weight (kg)	61.5 ± 7.8	62.8 ± 9.0	59.4 ± 5.0	0.317
BMI (kg/m^2^)	21.6 ± 5.2	24.1 ± 2.8	22.8 ± 1.2	0.103
Waist circumference (cm)	80.6 ± 5.6	84.4 ± 10.1	75.2 ± 22.0	0.157
Calf circumference (cm)	36.3 ± 2.7	37.1 ± 3.9	35.2 ± 3.4	0.240
Height (cm)	164.9 ± 6.9	161.0 ± 7.3	161.6 ± 5.7	0.083
Alanine transaminase (U/L)	20.4 ± 8.2	24.4 ± 22.6	17.7 ± 9.6	0.422
Triglycerides (mmol/L)	1.2 ± 0.4	1.5 ± 1.0	1.6 ± 0.9	0.374
Total cholesterol (mmol/L)	4.9 ± 0.7	5.2 ± 0.7	5.4 ± 1.6	0.196
Creatinine (μmol/L)	71.3 ± 18.2	68.4 ± 19.0	74.1 ± 17.6	0.636
Urea (mmol/L)	5.1 ± 1.2	5.7 ± 1.6	6.1 ± 1.5	0.126
Systolic blood pressure (mmHg)	131.6 ± 5.1	135.0 ± 4.5	132.6 ± 5.1	0.199
Diastolic blood pressure (mmHg)	78.4 ± 4.0	76.8 ± 7.6	78.4 ± 5.3	0.707
Hemoglobin (g/L)	142.9 ± 7.6	138.9 ± 11.2	137.3 ± 10.4	0.357
Fasting blood glucose (mmol/L)	5.6 ± 1.2	5.6 ± 0.9	5.3 ± 0.9	0.575
Uric acid (μmol/L)	304.0 ± 63.7	358.5 ± 48.9	329.6 ± 64.1	0.818
ASMI (kg/m^2^)	6.0 ± 1.2	5.9 ± 1.0	5.9 ± 0.8	0.532
Left upper limb muscle mass (kg)	1.9 ± 0.5	1.6 ± 0.4	1.8 ± 0.4	0.033
Right upper limb muscle mass (kg)	1.9 ± 0.6	1.9 ± 0.4	2.0 ± 0.5	0.352
Left lower limb muscle mass (kg)	6.1 ± 1.0	6.0 ± 1.2	5.8 ± 1.2	0.641
Right lower limb muscle mass(kg)	6.2 ± 1.0	5.9 ± 1.2	5.7 ± 1.2	0.270
Grip strength (kg)	30.1 ± 13.2	26.2 ± 12.2	23.3 ± 3.4	0.158
6‐m walking speed (m/s)	1.2 ± 0.2	1.2 ± 0.2	1.1 ± 0.2	0.186
Chair stand test (s)	8.8 ± 1.6	8.7 ± 1.7	8.8 ± 1.7	0.970
SPPB score	8.8 ± 2.1	7.6 ± 2.3	8.4 ± 2.0	0.094
Physical activity level (MET‐min/week)	1370.4 ± 121.7	1351.5 ± 130.4	1320.6 ± 95.1	0.954

After 6 months of intervention, dietary survey results indicated that there were no statistically significant differences among the three groups in the changes of total energy, dietary energy, carbohydrate, fat, and protein intake (*p* > 0.05). The actual supplemental protein intake was 15.2 ± 0.3 g/d in the WP group and 15.8 ± 0.4 g/d in the YP group, whereas no supplement was provided to the control group (Table 2).

**TABLE 2:** Changes in energy and macronutrient intake in 92 Chinese older adults at baseline and after 6 months.

	WP group (*n* = 31)			YP group (*n* = 30)			Control group (*n* = 31)			*p*
	Week 1	Week 24	Difference	Week 1	Week 24	Difference	Week 1	Week 24	Difference	
Dietary energy (kcal/d)	1512.2 ± 54.4	1475.5 ± 48.9	−36.7 ± 17.0	1542.8 ± 57.0	1331.3 ± 31.5	−211.5 ± 32.8	1482.4 ± 23.9	1401.8 ± 41.7	−80.7 ± 97.2	0.547
Dietary carbohydrates (g/d)	160.5 ± 7.9	155.7 ± 13.5	−4.8 ± 105.9	180.4 ± 9.4	147.9 ± 6.4	−32.5 ± 119.7	162.8 ± 5.1	138.4 ± 9.5	−24.4 ± 67.6	0.558
Dietary fat (g/d)	70.9 ± 13.4	66.6 ± 5.7	−4.3 ± 5.9	65.4 ± 12.4	57.4 ± 13.6	−8.0 ± 28.0	66.9 ± 3.3	70.1 ± 7.9	3.1 ± 5.9	0.401
Dietary protein (g/d)	58.1 ± 22.2	63.4 ± 2.2	5.3 ± 4.6	58.7 ± 2.3	55.9 ± 18.7	−2.3 ± 26.5	57.2 ± 18.6	54.4 ± 9.2	−2.8 ± 18.5	0.362
Protein supplement (g/d)	—	15.2 ± 0.3	—	—	15.8 ± 0.4	—	—	—	—	—
Total energy (kcal/d)	1512.2 ± 42.4	1549.3 ± 87.3	37.1 ± 625.2	1542.8 ± 57.0	1412.8 ± 38.8	−130.0 ± 733.6	1482.4 ± 43.9	1401.8 ± 41.7	−80.7 ± 476.2	0.581

*Note:* *p* values are for comparisons of pre–post differences in each outcome across the three groups.

No significant difference in the change of physical activity level was observed among the groups either (specific changes: whey protein group +33.0 ± 77.5, yeast protein group +4.2 ± 53.3, control group −72.1 ± 56.3 MET‐min/week; *p* = 0.478), indicating a negligible influence of the exercise factor on the outcomes (Table 3).

**TABLE 3:** Changes in physical activity levels in 92 Chinese older adults after 6 months.

	WP group (*n* = 31)		YP group (*n* = 30)		Control group (*n* = 31)		*p*
	Week 24	Difference	Week 24	Difference	Week 24	Difference	
Physical activity level (MET‐min/week)	1420.8 ± 127.3	33.0 ± 77.5	1374.6 ± 114.4	4.2 ± 53.3	1248.5 ± 92.9	−72.1 ± 56.3	0.478

*Note:* *p* values are for comparisons of pre–post differences in each outcome across the three groups.

Significant differences in body weight changes were observed among the groups (*p* = 0.048, ANCOVA). The whey protein (WP) group exhibited an average reduction of 5.4 kg, the yeast protein (YP) group showed a decrease of 0.9 kg, and the control group demonstrated an increase of 1.9 kg (Table 4). To assess the robustness of this finding, we conducted a sensitivity analysis excluding two participants in the WP group with extreme weight loss (> 10 kg); the between‐group difference remained statistically significant, with the WP group showing a mean reduction of 4.1 kg (*p* = 0.048). Verification procedures ensured data reliability: (1) fasting body weight was measured using the same calibrated electronic scale (MSG003), with the mean of three consecutive measurements recorded; and (2) double data entry achieved 100% consistency. No statistically significant differences were found in changes in body mass index (BMI), waist circumference, or calf circumference (all *p* > 0.05). Among the laboratory parameters, only the change in total cholesterol reached statistical significance between groups (*p* = 0.030). No significant differences were observed for changes in other parameters, including triglycerides, creatinine, urea, blood pressure, hemoglobin, fasting blood glucose, and uric acid (all *p* > 0.05).

**TABLE 4:** Changes in body measurements and laboratory indicators in 92 Chinese older adults after 6 months.

	WP group (*n* = 31)		YP group (*n* = 30)		Control group (*n* = 31)		*p*
	Week 24	Difference	Week 24	Difference	Week 24	Difference	
Weight (kg)	58.1 ± 8.6	−5.4 ± 10.5	62.5 ± 7.5	−0.9 ± 11.3	61.6 ± 7.6	1.9 ± 4.7	0.048
BMI (kg/m^2^)	21.9 ± 2.1	0.1 ± 4.6	23.0 ± 2.2	−1.1 ± 3.3	23.7 ± 2.1	0.4 ± 1.1	0.345
Waist circumference (cm)	78.2 ± 4.3	−1.6 ± 4.4	80.7 ± 6.0	−1.9 ± 10.4	82.8 ± 12.9	4.8 ± 26.0	0.497
Calf circumference (cm)	34.3 ± 2.2	−2.0 ± 3.2	36.0 ± 2.9	−0.9 ± 4.9	36.8 ± 4.3	0.3 ± 1.6	0.208
Alanine transaminase (U/L)	19.4 ± 11.4	−2.5 ± 15.2	18.2 ± 6.3	−8.0 ± 26.5	19.0 ± 9.3	0.2 ± 9.7	0.455
Triglycerides (mmol/L)	1.2 ± 0.5	−0.1 ± 0.4	1.3 ± 0.5	−0.3 ± 1.0	1.6 ± 0.9	−0.2 ± 0.9	0.657
Total cholesterol (mmol/L)	4.7 ± 0.8	−0.2 ± 0.6	4.8 ± 1.3	−0.2 ± 0.9	5.4 ± 0.9	0.3 ± 2.9	0.030
Creatinine (μmol/L)	75.0 ± 18.4	4.1 ± 31.1	71.0 ± 20.9	0.3 ± 35.9	65.1 ± 17.3	−9.5 ± 16.7	0.351
Urea (mmol/L)	5.9 ± 1.4	1.0 ± 1.8	5.9 ± 1.5	0.2 ± 2.5	5.7 ± 1.5	−0.5 ± 1.8	0.095
Systolic blood pressure (mmHg)	136.3 ± 16.9	3.7 ± 20.4	134.9 ± 8.6	−4.7 ± 4.7	133.5 ± 5.1	−1.1 ± 4.0	0.281
Diastolic blood pressure (mmHg)	76.2 ± 9.1	−3.5 ± 11.7	76.9 ± 7.1	1.9 ± 9.2	78.0 ± 5.7	−0.1 ± 5.7	0.342
Hemoglobin (g/L)	138.9 ± 7.6	−4.5 ± 3.2	137.5 ± 7.2	−1.4 ± 2.8	138.8 ± 161.9	2.6 ± 7.3	0.789
Fasting blood glucose (mmol/L)	5.6 ± 1.2	0.3 ± 0.1	5.7 ± 1.4	0.2 ± 1.4	5.3 ± 1.0	0.1 ± 2.7	0.429
Uric acid (μmol/L)	275.1 ± 82.0	−28.9 ± 19.8	334.5 ± 53.7	−23.9 ± 36.3	322.2 ± 64.8	−7.4 ± 28.0	0.345

*Note:* *p* values are for comparisons of pre–post differences in each outcome across the three groups.

The DXA results showed no significant differences among the three groups in changes in appendicular skeletal muscle mass (ASM) or appendicular skeletal muscle mass index (ASMI) (*p* = 0.117) (Table 5).

**TABLE 5:** Changes in lean body mass (kg) in Chinese older adults after 6 months.

	WP group (*n* = 31)		YP group (*n* = 30)		Control group (*n* = 31)		*p*
	Week 24	Difference	Week 24	Difference	Week 24	Difference	
ASMI(kg/m^2^)	6.0 ± 1.0	0.0 ± 2.9	5.9 ± 0.8	0.0 ± 2.4	5.9 ± 0.7	−0.4 ± 2.0	0.117
Left upper limb muscle mass (kg)	1.9 ± 0.6	0.0 ± 0.7	1.9 ± 0.7	0.3 ± 0.7	1.9 ± 0.5	0.1 ± 0.4	0.139
Right upper limb muscle mass (kg)	2.1 ± 0.6	0.2 ± 0.7	2.0 ± 0.7	0.1 ± 0.7	2.0 ± 0.5	0.0 ± 0.4	0.466
Left lower limb muscle mass (kg)	6.1 ± 1.3	0.0 ± 1.8	5.7 ± 1.9	−0.2 ± 1.8	5.7 ± 1.1	0.1 ± 1.4	0.820
Right lower limb muscle mass(kg)	6.1 ± 1.3	0.1 ± 1.9	5.8 ± 1.9	0.1 ± 1.6	5.7 ± 1.0	0.0 ± 1.3	0.891

*Note:* *p* values are for comparisons of pre–post differences in each outcome across the three groups.

Regarding muscle strength and physical performance, the between‐group differences in the change of handgrip strength were not significant (*p* = 0.135). A significant difference was observed in the change of 6‐m walking speed (*p* = 0.003), with the whey protein (WP) group showing an increase of 0.1 m/s, the yeast protein (YP) group an increase of 0.2 m/s, and the control group a decrease of 0.1 m/s. No significant difference was found in the change of chair stand test time (*p* = 0.581). A significant difference was noted in the change of SPPB total score (*p* = 0.002), with the WP group improving by 0.3 points, the YP group by 0.9 points, and the control group declining by 1.7 points. In summary, daily supplementation with whey protein or yeast protein was associated with improvements in 6‐m walking speed and SPPB score compared to the control group, whereas effects on handgrip strength and chair stand test were limited (Table 6).

**TABLE 6:** Changes in muscle strength and physical performance in 92 Chinese older adults.

	WP group (*n* = 31)		YP group (*n* = 30)		Control group (*n* = 31)		*p*
	Week 24	Difference	Week 24	Difference	Week 24	Difference	
Grip strength (kg)	27.0 ± 10.7	−5.0 ± 11.3	28.8 ± 11.2	0.7 ± 8.2	21.8 ± 2.6	−0.8 ± 2.7	0.135
6‐m walking speed (m/s)	1.3 ± 0.3	0.1 ± 0.2	1.3 ± 0.3	0.2 ± 0.2	1.0 ± 0.1	−0.1 ± 0.1	0.003
Chair stand test (s)	9.1 ± 1.5	0.1 ± 2.1	8.5 ± 1.4	−0.2 ± 0.6	8.9 ± 1.8	0.1 ± 0.4	0.581
SPPB score	9.4 ± 1.9	0.3 ± 2.9	8.4 ± 2.2	0.9 ± 2.7	6.8 ± 2.1	−1.7 ± 2.9	0.002

*Note:* *p* values are for comparisons of pre–post differences in each outcome across the three groups.

## 4 Discussion

Protein sources are diverse, including animal protein, plant protein, microbial protein, and others. Animal protein is typically a complete protein but contains higher levels of saturated fat and cholesterol. The effects of whey protein, or whey protein combined with resistance exercise, on skeletal muscle are well established (Cuyul‐Vásquez et al. 2023; Park et al. 2019). The role of soy protein, as a plant‐based protein source, in sarcopenia has also been investigated (Coelho‐Júnior et al. 2022; Lee et al. 2022; Yang et al. 2020). Microbial protein (yeast protein) represents an emerging and environmentally friendly protein source. This study aimed to investigate the effects of yeast protein compared to whey protein on skeletal muscle health.

This 6‐month randomized controlled trial systematically compared the effects of WP and YP supplementation on a comprehensive set of health indicators in Chinese older adults. The key findings demonstrate that, compared to the non‐supplemented control group, both protein interventions led to significant and clinically relevant improvements in physical function, specifically in 6‐m walking speed and SPPB scores. Beneficial effects were also noted in body weight and total cholesterol. In contrast, no significant between‐group differences were found for changes in handgrip strength, chair stand test time, or most other parameters.

### 4.1 Changes in Skeletal Muscle Mass, Strength, and Function

One of the most significant findings of this study is the effect of protein supplementation on skeletal muscle function indicators: an improvement in SPPB scores suggests enhanced balance and walking speed in older adults, leading to increased independence in daily living. Improved walking speed may reflect better neuromuscular efficiency, balance, and lower‐limb coordination—components that contribute to mobility even in the absence of measurable gains in muscle mass or maximal strength. Numerous studies have confirmed the benefits of whey protein intervention on skeletal muscle function. A meta‐analysis exploring the relationship between protein intake and physical function in older adults, which included 22 cross‐sectional studies involving 11,332 participants, found that protein intake (including whey protein) above the Recommended Dietary Allowance (RDA) was associated with better SPPB scores and faster walking speeds (Coelho‐Júnior et al. 2022). Improvements in both SPPB and walking speed are associated with a better quality of life (Pavasini et al. 2016). The improvements in SPPB score and walking speed were most pronounced in the yeast protein group, suggesting that microbial protein may be particularly effective in enhancing physical function in older adults. The underlying reason may be that, although yeast protein and whey protein originate from different sources, yeast protein ensures both quantity (containing 80 g of protein per 100 g) and quality of protein. Notably, yeast protein is rich in branched‐chain amino acids (Table S1), with leucine content approaching that of whey protein (9.9 vs. 11.1 g per 100 g). Among all amino acids, leucine is considered the most potent stimulator of protein synthesis (Cruz‐Jentoft et al. 2019). Leucine is a crucial regulator of muscle protein anabolism; it can trigger targets in the mammalian target of rapamycin (mTOR) pathway and inhibit proteases, thereby maintaining muscle mass (Solerte et al. 2008). Therefore, yeast protein has effects comparable to whey protein on skeletal muscle function in older adults.

However, a finding warranting further investigation is that although SPPB scores and walking speed showed significant improvement, no concurrent significant increase in appendicular skeletal muscle mass was observed. This suggests that the benefits of protein supplementation may be prioritized in skeletal muscle function rather than in skeletal muscle mass. Possible explanations include: (1) Measurement sensitivity: Compared to whole‐body muscle mass measured by dual‐energy X‐ray absorptiometry (DXA), SPPB and walking speed are comprehensive indicators reflecting overall physical function and mobility in the elderly, which are more sensitive to subtle, daily‐life‐related changes. (2) Intervention duration: The average age of this cohort was 73.2 years, characterized by higher anabolic resistance. A 6‐month intervention may be sufficient to induce adaptive changes in the neuromuscular system and physical function, but significantly reversing age‐related muscle loss (sarcopenia) likely requires a longer duration (≥ 12 months) or higher‐intensity combined interventions (e.g., incorporating resistance exercise). The results of this study showed that although 6‐m walking speed and SPPB scores significantly improved in the protein groups, no concurrent changes in lean body mass were observed. This dissociation between functional improvement and stable muscle mass aligns with prior observations (Chang and Chiu 2020; Chiu et al. 2018). Early research has found that grip strength declines faster (*β* = 0.809) than upper limb skeletal muscle mass (*β* = −0.592) with increasing age in older men. Similarly, walking speed declines faster than lower limb skeletal muscle mass in both older men (*β* = 0.683 vs. *β* = 0.442) and women (*β* = 1.00 vs. *β* = 0.461) (Bai et al. 2016), indicating a discrepancy between changes in skeletal muscle mass and function.

It is particularly noteworthy that no significant increase in grip strength was observed in the protein groups. This finding seems contradictory to the potent anabolic properties of whey protein. Several specific factors may explain this: First, the study population had a relatively high average age (73.2 years), where muscle protein anabolic resistance may be more pronounced, limiting the effect of nutritional supplementation alone on upper limb strength. Second, grip strength is an indicator of localized muscle strength, and its changes are influenced by factors such as baseline hand function, participant motivation, and effort during measurement, leading to higher variability. Finally, improvements in physical function (e.g., walking speed, balance) primarily rely on lower limb and core muscle groups, and the functional benefits of protein supplementation for these muscle groups may not be fully captured by handgrip dynamometry. Future studies incorporating more comprehensive strength tests (e.g., isokinetic knee extension strength) would help address this issue.

### 4.2 Body Weight Changes and Preservation of Muscle Mass

The study observed that the protein supplementation groups (whey protein and yeast protein groups) showed advantages in weight management, but no significant change in skeletal muscle mass was noted, which is consistent with previous research findings (Nilsson et al. 2024). Given that the 24‐h dietary recall method has limited sensitivity for detecting subtle energy changes (< 150 kcal/day), the observed weight loss might reflect a mild energy deficit induced by increased protein satiety that was not fully captured by dietary records. Potential mechanisms include: (1) The higher thermic effect of protein increases energy expenditure. (2) Significant changes in body weight may be related to protein supplementation, as protein can increase satiety and reduce energy intake. (3) Enhancing satiety by modulating appetite‐related hormones (e.g., GLP‐1) (Westerterp‐Plantenga 2008). (4) Improving muscle protein synthesis efficiency and reducing age‐related muscle loss. Leucine in protein is a crucial regulator of muscle protein anabolism and also plays a role in reducing age‐related muscle loss. Although the changes in body weight were statistically significant, their clinical significance requires cautious interpretation. Weight loss in the elderly population may have dual implications: for those who are overweight or obese, moderate weight loss may be beneficial; however, for individuals with low baseline weight or at risk of malnutrition, weight loss may indicate adverse health outcomes. Therefore, it is recommended that future research incorporate clinical endpoint events (e.g., falls, hospitalization rates, and quality of life scores) to further validate the clinical value of these changes. It is important to note that the WP group showed an average weight loss of 5.4 kg, whereas DXA‐measured muscle mass, waist circumference, and 24‐h dietary recall did not show concurrent changes. A review of original records and statistical procedures ruled out weighing and data entry errors; this phenomenon aligns with observations from previous protein interventions lasting ≥ 6 months in similar populations (Chiu et al. 2018). Therefore, we consider this “weight loss with stable muscle mass” phenotype physiologically plausible, and the limitations of dietary recall in detecting subtle energy changes have been addressed in the limitations section.

### 4.3 Laboratory Indicators

This study observed that after the 6‐month intervention, total cholesterol decreased by an average of 0.15 mmol/L in the WP group and 0.18 mmol/L in the YP group, with a statistically significant between‐group difference (*p* = 0.030). However, we acknowledge that our lipid profile assessment was incomplete as low‐density lipoprotein cholesterol (LDL‐C) and high‐density lipoprotein cholesterol (HDL‐C) were not measured; therefore, no conclusions can be drawn regarding lipoprotein‐specific effects or cardiovascular risk implications. This indicates that yeast protein is a potential tool for cholesterol management, and these mechanisms are supported by multiple studies (Lærke et al. 2014; Mitchelson et al. 2022; Whitehead et al. 2014). In elderly populations, a TC reduction of 0.1–0.2 mmol/L has been reported to reduce the risk of cardiovascular events by approximately 5%–7% (Cholesterol Treatment Trialists' (CTT) Collaboration 2019; Law et al. 2003; Prospective Studies Collaboration 2007). We hypothesize that this change may be related to the following factors: (1) Protein supplementation may have partially displaced foods high in saturated fat or dietary cholesterol, potentially contributing to the observed reduction in total cholesterol; (2) yeast protein contains a small amount of dietary fiber, which may mildly modulate gut microbiota or bile acid metabolism. Given that LDL‐C and HDL‐C were not assessed, no conclusions can be drawn regarding lipoprotein‐specific effects. This suggests that yeast protein may exert a mildly beneficial effect on lipid metabolism by improving total cholesterol, but its specific effects on LDL‐C or HDL‐C require further verification in studies with complete lipid profiles. Given the incomplete lipid data, we recommend that future research include complete lipid profiles (LDL‐C, HDL‐C) to further validate the metabolic effects of protein supplementation.

### 4.4 Study Implications, Limitations, and Future Directions

Yeast protein is derived from *Saccharomyces cerevisiae* through fermentation technology. Its production does not rely on traditional agriculture or animal husbandry, offering high efficiency, low cost, readily available raw materials, mature processes, and environmental friendliness. As an emerging high‐quality protein source, yeast protein is nutritious and safe. Furthermore, cost‐effectiveness is a profound consideration when selecting protein supplements. The market price of yeast protein is generally around 200 CNY/kg (approximately 28 USD/kg). In comparison, ordinary whey protein powder typically costs between 300 and 500 CNY/kg (approximately 42–69 USD/kg) (taobao.com). On the basis of typical market prices, the estimated daily cost for a 20 g supplement is approximately 4 CNY (0.56 USD) for yeast protein versus 6–10 CNY (0.83–1.39 USD) for whey protein. To our knowledge, this is among the first studies to systematically compare the effects of whey protein and yeast protein in an elderly Chinese population, providing a scientific basis for developing targeted nutritional intervention strategies. A daily protein supplementation dose of 15–20 g demonstrated favorable effects in this study, consistent with previous findings (Huang et al. 2021; Li et al. 2021).

However, this study has several limitations: (1) The sample size, although adequate for the primary analysis, precluded meaningful subgroup analyses (e.g., by sex or baseline nutritional status). (2) The 6‐month intervention period may be insufficient to observe long‐term effects. (3) Dietary assessment using the 24‐h recall method has limited sensitivity for detecting “subtle” changes (< 150 kcal/d) in total energy and macronutrient intake. This may underestimate the mild energy deficit induced by protein supplementation, thereby failing to fully account for the observed weight loss.

### 4.5 Future Directions

(1) Investigate the differential effects of various protein sources (animal, plant, and microbial) and their potential synergistic interactions. (2) Explore the synergistic effects of protein supplementation combined with exercise training, including both resistance and aerobic exercise. (3) Conduct research on personalized protein supplementation regimens for populations with different characteristics (e.g., different sexes, stages of sarcopenia, varying nutritional statuses). (4) Evaluate the effects of long‐term (≥ 12 months) interventions. (5) Perform gut microbiota analysis to explore the mechanistic basis for the effects of yeast protein on skeletal muscle. (6) Validating the weight loss mechanism through longer‐term studies with more precise body composition assessment.

## Author Contributions

Study design and manuscript preparation: Bai Huijing, Sun Jianqin, and Ma JiangChen. Data Collection: Shen Lei, Zhao Fang, Li Si, Tan Li, Gong Qinqing, and Gu Dongmei from the Caohejing Community Health Service Center, Xuhui District. Data Processing and Analysis: Bai Huijing.

## Funding

This work was supported by Angel Nutritional Fund, AF2021001.

## Supporting information


**Table S1:** The nutritional components of whey protein and yeast protein used in the randomized controlled clinical trial.

## Data Availability

The data that support the findings of this study are available from the corresponding author upon reasonable request. Due to privacy and ethical restrictions related to the clinical trial protocol (ChiCTR2200057116), the individual participant data are not publicly available to protect participant confidentiality.
