# Supplementary material for: The Impact of Oral Whey Protein and Yeast Protein Supplementation for 6 Months on Skeletal Muscle Mass, Strength, and Function in the Elderly: A Randomized, Controlled, Parallel Study
Source: Food Sci Nutr. 2026 Mar 13;14(3):e71552. doi: 10.1002/fsn3.71552 (PMC13093566; doi:10.1002/fsn3.71552)
Supplement: Supplementary file 1 — Table S1: The nutritional components of whey protein and yeast protein used in the randomized controlled clinical trial. [file FSN3-14-e71552-s001.docx]

Supplementary Table 1: The nutritional components of whey protein and yeast protein used in the randomized controlled clinical trial

|  | WP  (per 100 g) | YP  (per 100 g) | WP  (per 20 g) | YP  (per 20 g) |
| --- | --- | --- | --- | --- |
| Energy（KJ） | 1570 | 1731 | 314 | 346 |
| Carbohydrates (g) | 2.9 | 0.6 | 0.58 | 0.12 |
| Fat(g) | 4.2 | 8.7 | 0.84 | 1.74 |
| Protein(g) | 77.5 | 80.0 | 15.5 | 16.0 |
| Dietary Fiber(g) | 5.6 | 4.8 | 1.12 | 0.96 |
| Amino Acids(g) |  |  |  |  |
| Alanine | 5.5 ± 0.1 | 6.1 ± 0.1 | 1.10 ± 0.02 | 1.22 ± 0.02 |
| Arginine | 3.6 ± 0.1 | 8.4 ± 0.1 | 0.72 ± 0.02 | 1.68 ± 0.02 |
| Asparagine | 11.2 ± 0.3 | 9.5 ± 0.1 | 2.24 ± 0.06 | 1.90 ± 0.02 |
| Glutamic acid | 17.9 ± 0.3 | 10.0 ± 1.0 | 3.58 ± 0.06 | 2.00 ± 0.20 |
| Glycine | 2.2 ± 0.1 | 5.1 ± 0.1 | 0.44 ± 0.02 | 1.02 ± 0.02 |
| Histidine | 2.4 ± 0.1 | 3.3 ± 0.1 | 0.48 ± 0.02 | 0.66 ± 0.02 |
| Isoleucine | 6.6 ± 0.1 | 6.7 ± 0.1 | 1.32 ± 0.02 | 1.34 ± 0.02 |
| Leucine | 11.1 ± 0.3 | 9.9 ± 0.1 | 2.22 ± 0.06 | 1.98 ± 0.02 |
| Lysine | 8.1 ± 0.2 | 7.5 ± 0.1 | 1.62 ± 0.04 | 1.50 ± 0.02 |
| Phenylalanine | 4.3 ± 0.1 | 6.8 ± 0.1 | 0.86 ± 0.02 | 1.36 ± 0.02 |
| Proline | 6.3 ± 0.1 | 4.3 ± 0.1 | 1.26 ± 0.02 | 0.86 ± 0.02 |
| Serine | 6.3 ± 0.1 | 6.0 ± 0.1 | 1.26 ± 0.02 | 1.20 ± 0.02 |
| Tryptophan | 2.0 ± 0.2 | 1.5 ± 0.1 | 0.40 ± 0.04 | 0.30 ± 0.02 |
| Tyrosine | 4.4 ± 0.1 | 5.6 ± 0.0 | 0.88 ± 0.02 | 1.12 ± 0.00 |
| Valine | 6.2 ± 0.1 | 7.0 ± 0.1 | 1.24 ± 0.02 | 1.40 ± 0.02 |
